# MicroED for GPCRs

**DOI:** 10.1063/4.0000992

**Published:** 2025-10-27

**Authors:** Anna Shiriaeva

**Affiliations:** UCLA, Los Angeles, CA, USA

## Abstract

Microcrystal Electron Diffraction (MicroED) is an emerging CryoEM technique that enables high-resolution structure determination of biological molecules by analyzing electron diffraction patterns from single micro- or nanocrystals on EM grids. This approach offers distinct advantages for investigating challenging membrane protein targets.

G-protein-coupled receptors (GPCRs) constitute a large superfamily of membrane proteins that play a critical role in cellular signaling by transducing extracellular stimuli into intracellular responses. Due to their central role in physiological processes, GPCRs are among the most clinically relevant drug targets.

Crystallization of GPCRs is commonly performed in a lipidic cubic phase (LCP) to provide a stabilizing lipidic environment to membrane proteins during crystallization. However, GPCRs frequently form microcrystals in LCP that are too small for conventional X-ray crystallography. While these microcrystals can be studied using MicroED, the high viscosity of LCP presents a significant challenge for standard MicroED sample preparation, necessitating specialized approaches to enable structural analysis.

In developing an approach to target protein crystals within the lipidic cubic phase (LCP), we addressed several significant challenges, including the high viscosity of the LCP medium, the precise localization of microcrystals, and the milling of lamellae containing microcrystals within the frozen LCP. Our initial attempt to determine the structure of the adenosine A2A receptor from a single nanocrystal yielded a resolution of 2.8 Å. The total diffracting volume was less than 1 μm³ (0.2 × 2 × 2 μm), which is smaller than that achieved by any other method for GPCR structure determination to date. Subsequent improvements in sample preparation, data collection and electron detection resulted in a structure at 2.0 Å resolution, comparable to the structure obtained using X-ray free electron laser (XFEL) techniques.

We developed a robust approach that involves labeling proteins with an NHS-ester-linked fluorescent dye prior to crystallization. This method incorporates an integrated fluorescence light microscope (iFLM) within a focused ion beam and scanning electron microscope (FIB-SEM) to identify fluorescent-labeled GPCR microcrystals.

This approach was applied to the challenging G protein-coupled receptor (GPCR) target, vasopressin receptor 1B (V1B). V1B plays a crucial role in regulating the release of corticotropin and has been implicated in various physiological processes, including stress response and regulation of the hypothalamic-pituitary-adrenal axis.

Due to its membrane localization and structural complexity, V1B is considered a difficult target for structural analysis. By utilizing our optimized MicroED approach and overcoming the challenges posed by the lipidic cubic phase (LCP), we were able to solve the receptor structure at 3.2 Å resolution in the P1 space group from 14 crystalline lamellae. This work demonstrates the potential of MicroED to determine previously inaccessible GPCR structures.

This study advances the application of MicroED and plasma beam milling for GPCR structure determination. Our methodology can be adapted for the structural characterization of novel GPCRs and other challenging membrane proteins from microcrystals that would be inaccessible using conventional crystallographic techniques.

